# Case of Tracheoesophageal Fistula Formation as a Rare Complication of Antiangiogenic Tyrosine Kinase Inhibitor Therapy for Metastatic Hepatocellular Carcinoma

**DOI:** 10.7759/cureus.41783

**Published:** 2023-07-12

**Authors:** Oluwatayo Adeoye, Olga Kozyreva

**Affiliations:** 1 Medicine, St. Elizabeth's Medical Center, Brighton, USA; 2 Medical Oncology, Dana-Farber Cancer Institute, Brighton, USA

**Keywords:** esophageal stent, tracheoesophageal fistula, hepatocellular carcinoma, antiangiogenic tyrosine kinase inhibitors, cabozantinib

## Abstract

Treatment with antiangiogenic tyrosine kinase inhibitors (TKIs) has shown longer overall survival (OS) and progression-free survival (PFS) than with placebo in patients with advanced hepatocellular carcinoma (HCC) who have previously received systemic therapy. Unfortunately, TKIs are associated with some rare adverse events such as tracheoesophageal fistula formation (TEF). The common risk factors for TEF formation include radiation therapy, prior instrumentation of the esophagus/airway, surgery, and esophagitis. We present a case of a 64-year-old man with a history of HCC who developed TEF after three months of treatment with cabozantinib. Patients experiencing these events require prompt termination of the antiangiogenic TKI. Urgent intervention should be pursued to prevent respiratory failure. Clinicians should be aware of the potential adverse effects of antiangiogenic TKIs, especially in high-risk patients.

## Introduction

Hepatocellular carcinoma (HCC) accounts for 80% of all primary liver cancers and is categorized as one of the most common cancers worldwide although metastatic liver tumors are more frequently seen. Unfortunately, incidence and mortality rates keep rising. Following diagnosis, HCC has a median survival of six to 20 months and five-year survival of less than 10% [1]. HCC has a poor prognosis; hence treatment is requisite. Treatment with antiangiogenic tyrosine kinase inhibitors (TKIs) (e.g., cabozantinib) has shown longer overall survival (OS) and progression-free survival (PFS) than placebo in patients treated with systemic therapy and progression of disease was observed [2]. Although TKIs are beneficial, some potential life-threatening adverse events have been reported including GI and non-GI fistula formation (e.g., tracheoesophageal fistula). The culprit risk factors most involved include radiation therapy, prior instrumentation of the esophagus/airway, surgery, and esophagitis [3]. Patients experiencing this adverse effect require care urgently due to the high risk of respiratory failure from aspiration pneumonia. Most published data about TEFs are mostly from case reports. As the use of TKIs is increasing, we expect to see more of these rare and life-threatening adverse events. Herein, we present a case of a patient treated with cabozantinib with the subsequent development of TEF.

## Case presentation

**Figure 1:**
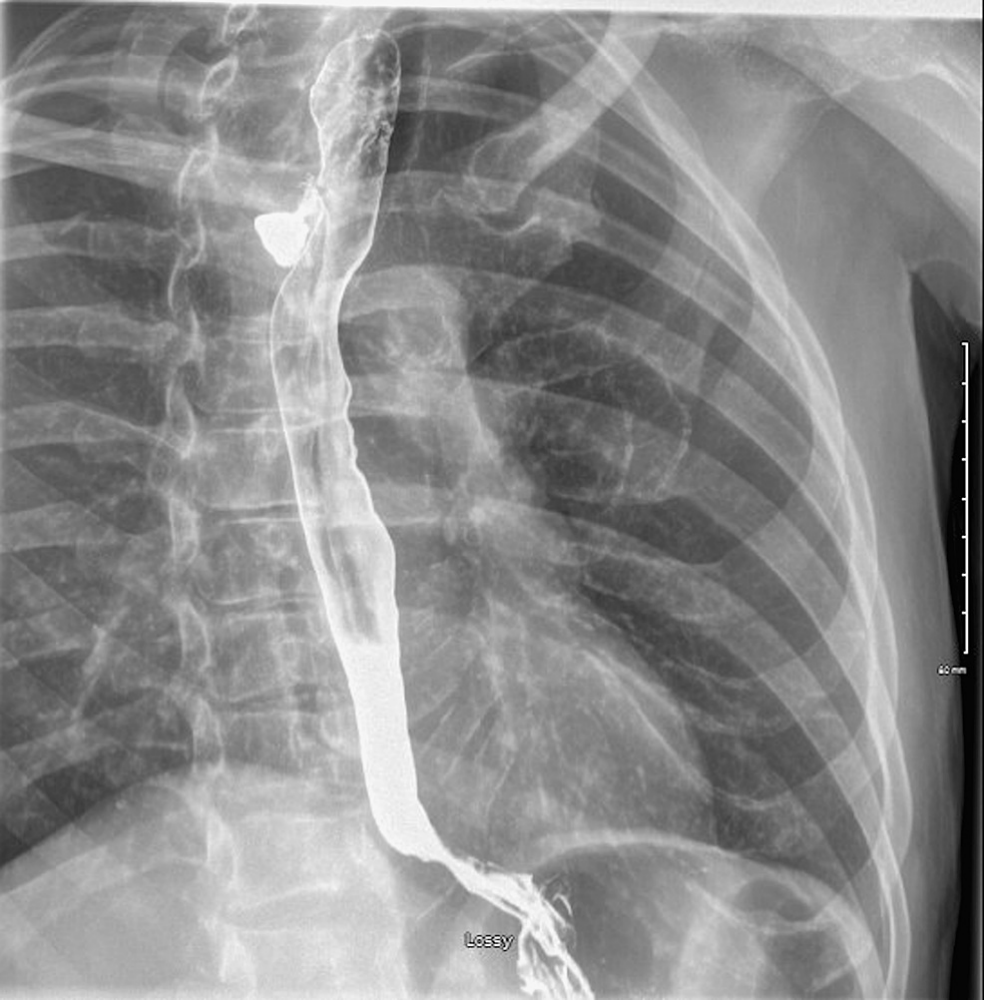
Barium esophagram showing a focus of contrast leak arising from the proximal thoracic esophagus extending into the right posterior mediastinum consistent with esophageal perforation

**Figure 2:**
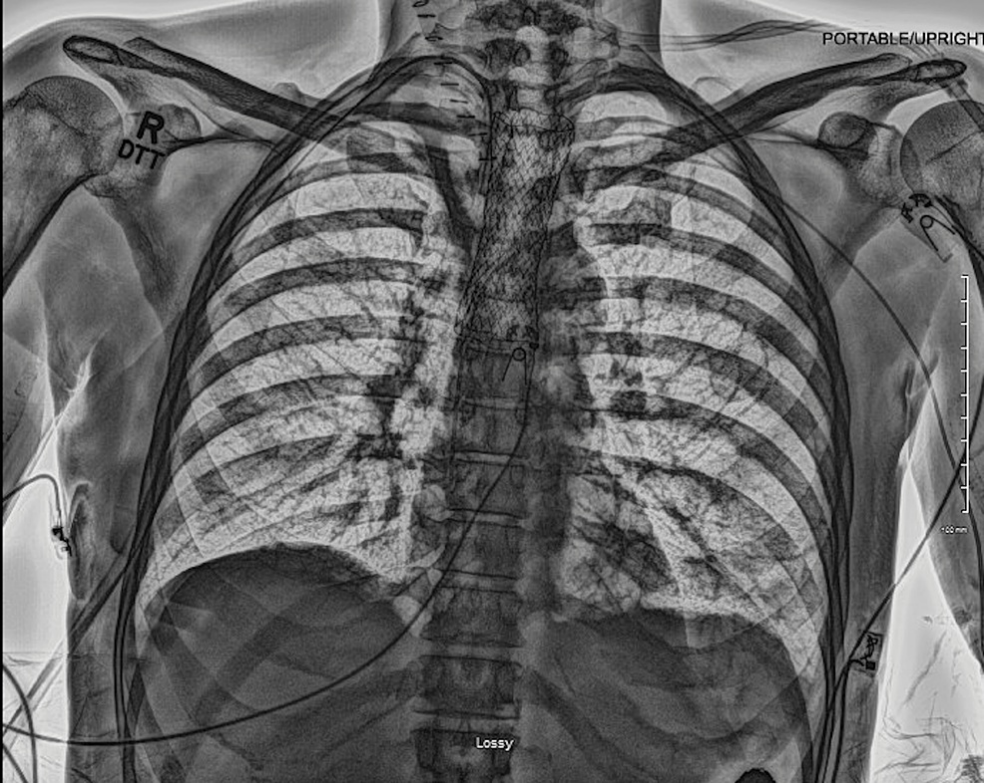
Chest X-ray showing a well-placed esophageal stent

**Figure 3:**
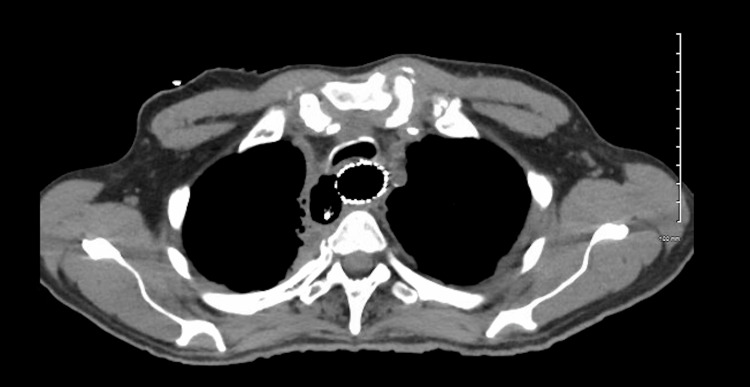
Computed tomographic images status-post esophageal stent placement

A 67-year-old man with a history of successfully treated chronic hepatitis C, active HCC treated with cabozantinib after lenvatinib failure, and poorly differentiated non-small cell lung cancer treated with chemoradiation and lung resection presented to the emergency department (ED) with fatigue, nausea unrelieved by antiemetics, chest pain, dysphagia, and regurgitation for several weeks accompanied by a 25-lb weight loss. His vitals were notable for tachycardia and his physical exam was unremarkable. EKG and laboratory values including troponins were unremarkable. Chest X-ray showed no acute infiltrates in the lungs and mediastinum. An esophagram showed a focus of contrast leak from the proximal thoracic esophagus extending into the right posterior mediastinum consistent with esophageal perforation (Figure 1). He was started on antibiotics, and thoracic surgery was consulted. Cabozantinib was stopped. Upper endoscopy revealed a posterolateral esophageal perforation at 18 cm from the incisors extending to 20 cm with evidence of debris beyond the perforation. There was evidence of esophagitis extending 3 cm proximal to the gastroesophageal junction (GEJ). Bronchoscopy revealed the presence of a fistula measuring about 1 cm on the right lateral border of the trachea at the junction of the membranous and cartilaginous portions. He was found to have a sizeable tracheoesophageal fistula requiring esophageal stent placement. Endoscopy was repeated to confirm successful stent placement. He also underwent right neck exploration of the mediastinum showing esophageal perforation in communication with the trachea. Purulent material was found, and a Blake drain was left in place in the cavity between the trachea and esophagus. The location of the tracheal defect was not amenable to stenting. Afterward, he was evaluated with a repeat esophagram (Figure 2) and chest CT (Figure 3), which showed no active extravasation of contrast in the region of the esophageal stent. He was started on a full-liquid diet, which he tolerated well with no symptoms. He was eventually discharged after his oral diet was restarted. A few months later, his drain fell out and he re-presented to the ED. A repeat chest CT was unremarkable. He was evaluated by thoracic surgery and discharged after that to follow up with oncology for possible initiation of yttrium-90 (^90^Y) radioembolization therapy.

## Discussion

In 2019, the United States Food and Drug Administration (FDA) approved cabozantinib for patients with HCC based on findings of CELESTIAL (Study of Cabozantinib (XL184) vs Placebo in Subjects With Hepatocellular Carcinoma Who Have Received Prior Sorafenib), a phase 3 trial that enrolled patients who progressed on systemic therapy for advanced/unresectable HCC. Cabozantinib showed better OS (10.2 vs 5.2 months) and PFS (5.2 vs 1.9 months) than placebo [2]. Cabozantinib, an orally bioavailable antiangiogenic TKI, inhibits vascular endothelial growth factor receptors (VEGF), which is important in the progression of hepatocellular carcinoma. These agents are designed to impair new blood vessel growth (angiogenesis) and delay wound healing, which in turn increases the risks of fistula formation [4].

This case report highlights the formation of TEF post-cabozantinib treatment for HCC. The risk factors most elucidated in studies include radiation therapy, prior instrumentation of the esophagus, surgery, and esophagitis [5]. The product warning label for cabozantinib reports non-gastrointestinal (GI) fistulas including tracheal/esophageal fistulas in 4% of treated patients with two fatal cases [6]. One study described aerodigestive fistula formation after treatment with cabozantinib, sunitinib, and lenvatinib for thyroid cancers. In that study, these risk factors were present [5]. In a phase II study in patients with salivary gland cancer (SGC), one out of six patients developed TEF in an area previously exposed to high-dose (70 Gy) radiotherapy. The time between radiotherapy and the start of cabozantinib was 51.1 months. TEF formation was after 7.8 months of cabozantinib initiation [3]. Another study showed the development of TEFs in two patients with lung cancer after bevacizumab treatment combined with chemoradiation [7]. TEF formation was also reported in five out of 43 patients with head and neck cancers treated with bevacizumab and chemoradiation in another study [8]. Bevacizumab is thought to exert similar effects on angiogenesis and wound repair as TKIs such as cabozantinib [4].

Our patient had a history of surgery (left lower lobe resection) and radiation for his prior lung cancer but described no fistula symptoms for years after these interventions. TEF formation has been reported years after radiotherapy in patients treated with antiangiogenic TKIs. Our patient’s symptoms developed three months after cabozantinib initiation compared to 7.8 months in the study with SGC patients [3]. In a phase 1 study of cabozantinib in thyroid cancers, one treatment-related death due to TEF was reported in a patient with prior history of mediastinal node resection and radiation. An esophageal stent was placed but, unfortunately, the patient developed aspiration pneumonia leading to death [9]. It is also important to mention that our patient failed treatment with lenvatinib, which has been implicated in TEF formation in other studies [3,10].

To prevent morbidity and mortality, it is important to identify patients at risk for this rare but potentially fatal adverse effect. It is also essential to continue studying antiangiogenic TKIs to find safer alternatives for patients at high risk for fistula formation. Further studies are needed to understand risk factors better and caution should be observed when using TKIs in patients with known risk factors. Preventive interventions to avoid TEF occurrence in patients treated with cabozantinib should also be studied to prevent this rare but life-threatening complication. Therapy should be discontinued in patients who develop TEFs, and stent placement should be considered [6,11]. In addition, the patient should be food-deprived until safe to introduce an oral diet.

Our patient showed no signs of repeat TEF after esophageal stent placement and after discontinuation of cabozantinib. This suggests that early diagnosis of TEF and immediate interventions to prevent fatal complications (e.g., aspiration pneumonia) are necessary for improving survival and quality of life.

## Conclusions

In conclusion, we present a case of TEF occurring as a complication of cabozantinib treatment. As these medications develop more of a role in cancer treatment, it is critical to understand their rare but life-threatening adverse effects. Patients with risk factors should be monitored closely upon initiation of antiangiogenic TKIs. Early diagnosis is important to prevent fatal complications and cabozantinib must be discontinued in these patients.
